# CYP17 MspA1 Gene Polymorphism and Breast Cancer Patients According to Age of Onset in Cancer Institute of Iran

**Published:** 2017-04

**Authors:** Elmira EBRAHIMI, Tayebeh SABOKBAR, Sharareh ESKANDARIEH, Vahideh PEYGHAMBARI, Reza SHIRKOOHI

**Affiliations:** 1. Group of Genetics, Cancer Research Center, Cancer Institute, Imam Khomeini Hospital Complex, Tehran University of Medical Sciences, Tehran, Iran; 2. Neurology & Neurosciences Research Center, Qom University of Medical Sciences, Qom, Iran; 3. Brain and Spinal Cord Injury Research Center, Neuroscience Institute, Tehran University of Medical Sciences, Tehran, Iran; 4. MS Research Center, Neurosciences Institute, Tehran University of Medical Sciences, Tehran, Iran

**Keywords:** Breast cancer, *CYP17* gene, Early onset breast cancer, Estrogen, Late-onset breast cancer

## Abstract

**Background:**

Exposure to endogenous hormones such as estrogen is known as a lifetime Breast Cancer (BC) risk factor. Polymorphisms in genes that are involved in the steroidogenic process, such as Cytochrome P450c17alpha (*CYP17)*, affect individuals’ susceptibility to BC. In Iran, the highest incident of BC is among young women. This study aimed to find prevalence of Single Nucleotide Polymorphisms (SNPs) in genes such as *CYP17* and significant correlation with age-oriented group of breast cancer.

**Methods:**

In 2016, a case series study was conducted on a total population of 205 patients suffering from breast cancer referred to Cancer Institute, Imam Khomeini Hospital Complex, Tehran, Iran. This population consisted of 104 cases less than 40 yr old and 101 cases over 40. The genotype variants of *CYP17 MspA1* were determined using PCR, followed by RFLP. The association of *CYP17 MspA1* polymorphisms with the risk of BC in two different age groups was evaluated by calculating odds ratio and 95% confidence intervals using unconditional logistic regression.

**Results:**

Carriers of at least one A2 allele may have higher risk of developing breast cancer at younger age compared to patients with A1/A1 genotype (Odds Ratio: 1.99, 95% Confidence Interval: 1.11–3.57, *P=*0.02).

**Conclusion:**

*CYP17*gene polymorphisms may have influence on the early onset of breast cancer.

## Introduction

Breast Cancer (BC) is the most prevalent malignancy and is known as the second leading cause of cancer death among women all around the world (1). In Iran, it has the highest cancer rate among women and accounts for 24.4% of all cancers affecting women (2, 3).

Mixture of genetic susceptibility and environmental exposure are widely accepted as causes of breast cancer. Over the last decades, several different factors such as early menarche, late menopause, post menopause obesity, hormone replacement therapy (HRT), and drinking alcohol have been identified and are strongly linked to increased risk of breast cancer (1, 4, 5).

As an environmental risk factor, exposure to endogenous hormones such as estrogen is known as the lifetime BC risk factor. Although the exact mechanism is not well understood, however, there are two proposed hypotheses. The first one is based on the intrinsic characteristic of the estrogen, as it is a cell proliferation driver. Increase in the number of cells can lead to increase in the number of errors during DNA replication and ultimately increase in the incident of random mutations. The second hypothesis explains that estrogen can be metabolized into quinone derivatives that are involved in DNA depurination which can make DNA prone to more errors and generate carcinogenic mutations (6, 7).

In addition, factors which have influence on the activity and/or metabolism of steroid hormones are of high importance since they potentially affect the incidence of breast cancer. A good example of this is the cytochrome P450c17alpha (*CYP17*) gene. The location of this gene is on chromosome 10 at q24.3 (8). It encodes steroid 17-alpha-hydroxylase, also known as steroid 17-alpha-monooxygenase. It performs both 17-alpha-hydroxylase and 17, 20-lyase activity. Functions of this enzyme allow the adrenal glands and gonads to synthesize both 17-alpha-hydroxylated glucocorticoids (via 17-alpha-hydroxylase activity) and sex steroids (via 17, 20-lyase activity) (9–12). The level of endogenous estrogen varies in different people, which is the result of polymorphism in the steroidogenic genes that *CYP17* is one of them (13, 14). Currently, three important polymorphisms have been identified in *CYP17* gene. One of which is a single base polymorphism (SNP) T-> C (rs743572) in the promoter region of *CYP17* gene. This specific SNP can create an additional SP1 binding site (CCACC box) which in turn may enhance the gene expression that ultimately may lead to elevated level of estrogen (15, 16).

In Iran, highest incident of BC is among women aged between 40 to 49 yr old. The early age breast cancer is associated with worse prognosis, rapid disease progression and poorer response to treatment necessitates early screening tests and treatments base on the age of the patients (2). Altogether, an unopposed prolonged lifetime exposure of estrogen enhances the risk of breast cancer (17, 18). Studying the prevalence of SNPs in genes such as *CYP17* shows a significant correlation with age oriented group of breast cancer patients that suggests a screening marker for risk group can be developed.

This study aimed to find prevalence of Single Nucleotide Polymorphisms (SNPs) in genes such as *CYP17* and significant correlation with age-oriented group of breast cancer.

## Materials and Methods

### Study population

In this case series project conducted in 2016, 205 individuals suffering from breast cancer referred to Imam Khomeini Hospital (Tehran, Iran) were studied. All the subjects included in this study gave their informed consent.

The Ethical Committee of the Tehran University of Medical Sciences approved this study. Patients were categorized according to the age of onset into two groups of younger onset (<40 yr) and elder onset (≥40 yr). All the patients’ breast cancers were confirmed based on their positive histopathology results. A validating questionnaire was used to collect demographic information including age at time of diagnosis, marital and occupational status, educational level, hometown and residence and clinical information such as tumor characteristics, tumor location, type of breast cancer, age at menarche (under 12, 12 yr old or more) and family history of cancer (first to third degree relatives with history of cancer).

### Blood Collection and DNA Extraction

Five milliliters of peripheral blood were collected from each patient and later used for DNA isolation. Genomic DNA extraction was performed using Gentra Puregene Blood Kit (Qiagen).

### Genotyping

Genotyping of patients was determined by polymerase chain reaction (PCR) followed by restriction fragment length polymorphism analysis (RFLP). Briefly, 50 ng of genomic DNA was used in PCR reaction containing 50 pmol of forwarding primer as *5′-*GGCTCCAGGAGAATCTTTC-*3′* and reverse primer as *5′*-GGGCCAAAACAAATAAGCTA-*3′* (19), 100umol of dNTP, 1× Taq buffer and 1 unit of Taq polymerase in total volume of 25*μl*. The PCR procedure was performed with initiation temperature of 94 °C for 5 min followed by 30 cycles of 94 °C for 1 min for denaturing, 57 °C for 1 min for annealing and 72 °C for 1 min for elongation time. The final cycle was followed by 72 °C for 5 min for extension. PCR products were digested with MspAI restriction enzyme (CutSmart, New England BioLab, USA) at 37 °C for 15 min for investigating the presence of A1 (not digested) and A2 (T->C with MspAI digestion recognition site) genotypes. Digested fragments were stained with RedSafe Nucleic Acid Staining Solution (iNtRON Biotechnology) and further analyzed on 3% agarose gel. Digestion of the mutant genotype creates 86 and 123 bp products, while an uncut product indicate the wild type genotype of 209 bp.

### Statistical Analysis

Data were registered into excel file and transferred to SPSS Ver. 16.0 (Chicago, IL, USA) for analysis. Student *t*-test was used for statistical analysis (*P*-value). The risk of breast cancer in either age subgroups of population was estimated by calculating Odds Ratio (*OR*). The *X^2^* test was done to evaluate the consistency of the frequency of *CYP17* genotypes variants with Hardy-Weinberg equilibrium.

## Results

Out of 205 patients participated in this study, 104 cases (50.7%) were younger than 40 yr old and 101 cases (49.3%) were older than 40.

Age range in young onset group was between 19 to 39 yr old and the average was 33.7 (±4.6 *s*tandard deviation). In late onset group, the age range was between 40 and 82 and was average of 51.4 (±9 *s*tandard deviation). Only 1 case (1%) was male and belonged to late onset group. Majority of patients were married, homemaker and educated in both young and late onset groups. Demographic data of 2 age subgroups of breast cancer patients has shown in Table 1.

**Table 1:** Demographic and disease condition data of 2 age subgroups of breast cancer patients

**Variables**	**Under 40** **Total=104** **n (%)**	**Over 40** **Total=101** **n (%)**
**Gender**		
Female	104 (100)	100 (99)
Male	0 (0)	1(1)
**Marital status**		
Single	15 (14)	4 (4)
Married	88 (85)	94 (93)
Missing	1 (1)	3 (3)
**Occupational level**		
Housewife	75 (72)	76 (75)
Working	24 (23)	24 (24)
Missing	5 (5)	1 (1)
**Educational status**		
Educated	91 (87)	78 (77)
Uneducated	5 (5)	17 (17)
Missing	8 (8)	6 (6)
**Hometown**		
Tehran	32 (31)	47 (46%)
Other cities	72 (69)	54 (53%)
**Residence**		
Tehran	51(49)	77 (76)
Other cities	53(51)	24 (24)
**Type of BC**		
Invasive ductal carcinoma	59 (57)	59 (58)
Other types	19 (18)	23 (23)
Missing	26 (25)	19 (19)
**Breast Involvement**		
Unilateral	100 (96)	91 (90)
Bilateral	4 (4)	9 (9)
Missing	0 (0)	1 (1)
**Tumor Location (Laterality)**		
Left side	48 (46)	53 (52)
Right side	48(46)	35 (35)
Both	4 (4)	11 (11)
Missing	4 (4)	2 (2)
**Age at Menarche**		
12 yr old or under	25 (24)	18 (18)
Over 12	51 (49)	53 (52)
Missing	28 (27)	30 (30)

In regards to menarche age, recorded data was available for 147 patients. The rest of the 58 patients were unable to recall their exact age at menarche (Table 1).

Concerning the family history of cancer, majority of patients in both subsets of population had no family history of cancer (Table 2).

**Table 2:** Family history of cancer of 205 patients included in this study

**Family History of Cancer**	**Under 40** **n (%)**	**Over 40** **n (%)**
**First Degree relative**		
1 person	16 (15)	22 (22)
2 persons	5 (5)	7 (7)
3 persons or more	0 (0)	4 (4)
No History	83 (80)	68 (67)
Missing	0 (0)	0 (0)
**Second Degree relative**		
1 person	19 (18)	17 (17)
2 persons	6 (6)	12 (12)
3 persons or more	3 (3)	1 (1)
No History	75 (72)	71 (70)
Missing	1 (1)	0 (0)
**Third Degree relative**		
1 person	18 (17)	18 (18)
2 persons	4 (4)	2 (2)
3 persons or more	1 (1)	1 (1)
No History	81 (78)	80 (79)
Missing	0 (0)	0 (0)

**Fig. 1:**
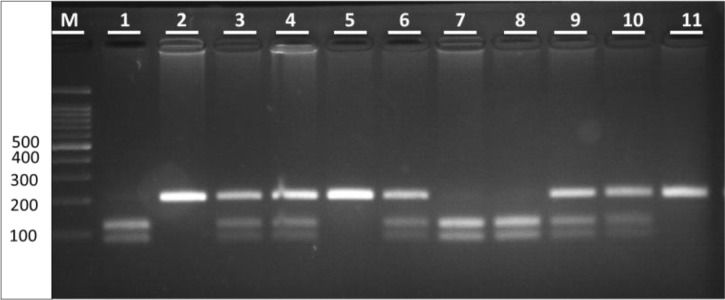
*CYP17 MspA1* digested products separated on 3% agarose gel electrophoresis Lane1 100bp ladder; Lane 2, 5 & 11 Homozygous A1 genotype (A1/A1); Lane 1, 3, 4, 6, 9 & 10 Heterozygous genotype (A1/A2); Lane 7 & 8 Homozygous A2 genotype (A2/A2).

In case of genotype variation in the whole population, 72 cases (35.1%) were A1/A1 (209 bp), 104 cases (50.7%) were A1/A2 (209, 123 and 86 bp) and 29 cases (14.1%) were A2/A2 (123 and 86 bp) (Fig. 1). The frequency of A1 and A2 alleles in the total population was 60.48% and 39.51%, respectively. The frequency of *CYP17* alleles in the total population did not deviate from Hardy-Weinberg equilibrium (*X^2^*=0.77, *P*=0.37).

Among the population of patients younger than 40 yr 27.8% had A1/A1, 55.7% had A1/A2, and 16.3% had A2/A2 genotype respectively (*X^2^*= 1.76, *P*=0.18) while it was 42.5%, 45.4% and 11.8% (*X^2^*=0.003, *P*=0.95) for elder group. No significant association was found between *CYP17* genotype variants and age of the patients. However, a significant correlation was found between the presence of at least one A2 allele and increased risk of breast cancer in patients age under 40 (*P=*0.02) (Table 3). In younger subset, the frequency of A1 and A2 allele was 55.76% and 44.23%, respectively while this was 65.34% and 34.65% for elder subset in that order. Comparing two subsets of groups in regards to laterality, type of BC and family history of cancer no significant association was found (Table 4). Regarding the menarche age, majority of young patients with at least one A2 allele had their first menstrual cycle at age 12 and this was statistically significant (*P=*0.04, OR: 0.35, 95% Confidence Interval: 0.12–0.98) (Table 4).

**Table 3:** Distribution of *CYP17 MSPA1* polymorphism in 2 subgroups of population

**Variables**	**Under 40** **n (%)**	**Over 40** **n (%)**	**OR**	**95%CI**	**P**
**Genotypes**					
Homozygous	29 (28)	43 (43)			0.88
A1/A1					
Heterozygous	58 (56)	46 (46)	2.101	0.875–5.046	0.97
A1/A2					
Homozygous	17 (16)	12 (12)	1.124	0.488–2.587	0.784
A2/A2					
**Combined polymorph**	75(72)	58 (57)	**1.996**	**1.116–3.572**	**0.02**
A1/A2 + A2/A2					

**Table 4:** Association between *CYP17 MspA1* polymorphism and potential BC risk factors such as laterality, type of breast cancer, family history of cancer and menarche age

**Variables**	**Patients Under 40**					**Patients over 40**				
	**Combined genotype A1/A2 & A2/A2 n (%)**	**Genotype A1/A1 n (%)**	**OR**	**95%CI**	***P* value**	**Combined genotype A1/A2 & A2/A2 n (%)**	**Genotype A1/A1 n (%)**	**OR**	**95%CI**	***P* value**
Unilateral	71 (71)	29 (29)	0	0	0.998	51 (56)	40 (44)	0.348	0.069–	0.203
Bilateral	4 (100)	0 (0)				7 (77.8)	2 (22.2)		1.769	
Invasive ductal carcinoma	47 (79.7)	12 (20.3)	2.848	0.939-	0.064	30 (50.8)	29 (49.2)	0.796	0.302-	0.644
Other types	8 (42.1)	11 (57.9)		8.640		14 (60.9)	9 (39.1)		2.098	
Family history of cancer	37 (69.8)	16 (30.2)	0.791	0.335-	0.594	38 (63.3)	22 (36.7)	2	0.892-	0.093
No family history of	38 (74.5)	13 (25.5)		1.871		20 (48.8)	21 (51.2)		4.486	
cancer										
Menarche age under 12	14 (56)	11 (44)	0.35	0.124-	**0.047**	9 (50)	9 (50)	0.963	0.330-	0.945
Menarche age 12 or over	40 (78.4)	11 (21.6)		0.984		28 (52.8)	25 (47.2)		2.806	

## Discussion

Endogenous exposure to circulating steroid hormones is known as a risk factor in developing breast cancer. There are different factors that have influence on the exposure to endogenous hormones and may affect susceptibility of the individuals to breast cancer. Polymorphisms in genes that are involved in the steroidogenic process, such as *CYP17*, are thought to be as one of these factors. Up to now, several studies have investigated the correlation of *CYP17 MspA1* polymorphism and risk of breast cancer but there is still a controversy in the results (20). In this case-series study, we demonstrated that carriers of at least one A2 allele might have higher risk of developing breast cancer at younger age compared to patients with A1/A1 genotype.

The frequencies of 34%, 52%, and 14% were reported for A1/A1, A1/A2, and A2/A2, respectively, in patients with polish origin (21). A prevalence of 41.5% for A1/A1, 47.4% was reported for A1/A2 and 11.1% for A2/A2 genotypes in Finnish population which were in accordance with our findings (22). Similar frequencies were also reported for different variants of *CYP17 MspA1* polymorphism (23).

Genotypes other than A1/A1 which may have more activity for *CYP17* enzyme for estrogen metabolism (A1/A2, A2/A2) were more frequent among younger breast cancer patients compared to older ones and this was statistically significant (*P*=0.02). Carriers of at least one A2 allele may have higher risk of developing breast cancer at younger age compared to patients with A1/A1 genotype (*OR:* 1.99, *95% CI:* 1.11–3.57). In 1996, the first research group brought up the association of A2 allele and increased risk of breast cancer (18). Higher risk of breast cancer was reported among younger patients who were Hetero- and Homozygote for A2 allele and our results are in line with their findings (19). Statistically significant association between A2 allele and early age breast cancer were reported (24). On the other hand, two meta-analyses, showed lack of correlation between *CYP17* polymorphisms and overall risk of breast cancer (20, 25–28). A reason that can be brought for the existing controversy is that different studies used different subgroups of populations defined by ethnicity, age (24, 29, 30), tumor stage/aggressiveness and menopausal status which may vary from one study to the other (31, 32). A second reason could be due to this fact that early onset of breast cancer is different with late onset in terms of pathological characteristics, diagnosis, and biological origin. This could explain the difference between 2 subgroups of population and controversy in different studies (19).

The relationship between estrogen exposure and risk of breast cancer were evaluated. Early menarche and late menopause, which means longer exposure to estrogen, increase the risk of breast cancer; however, in the present study, a significant relationship was found between young patients harboring A2 allele and late menarche. Several other studies including a meta-analysis also failed to show any association between *CYP17 MspA1* polymorphism and early menarche (33, 34). Twin and Family studies have shown that genetic factors account for 53–72% of menarche age variation (34). Thus, *CYP17* polymorphism may not be the only effect modifier in this regards and other genes may contribute to this trait variation.

Analyzing the *CYP17 MspA1* polymorphism in two subsets of population did not show any association with family history of cancer. Other genes are participating in steroidogenic pathways that may have greater roles in familial cases.

## Conclusion

Polymorphism in *CYP17* is possibly age specific and in addition to other risk factors may have influence on increased risk of breast cancer in early age. *CYP17 MspA1* polymorphism can be used as a potential genetic marker for identification of high-risk groups. However, a larger population is needed to confirm the major influence of *CYP17 MspA1* variants on early onset breast cancer among Iranian women.

## Ethical Consideration

Ethical issues (including plagiarism, obtaining informed consent, misconduct, data fabrication and/or falsification, double publication and/or submission, redundancy, etc.) have been completely observed by the authors.
